# Supplementary material for: Immediate and short-term effects of single-task and motor-cognitive dual-task on executive function
Source: PLoS One. 2023 Aug 16;18(8):e0290171. doi: 10.1371/journal.pone.0290171 (PMC10431647; doi:10.1371/journal.pone.0290171)
Supplement: Attachment — Submitted filename: response to reviewers.docx [file pone.0290171.s001.docx]

Dear reviewers:

Thanks very much for reviewing our manuscript timely and carefully. You have given us lots of good suggestions. We’ve revised our paper according to your comments, and the revised part was marked in red. Followed are our replies to your comments.

Reviewer#1:

Thank you again for your positive comments and valuable suggestions to improve the quality of our manuscript.

Reviewer#2:

Comment 1.the justification of the work is poor.it must be improved.

Answer: we agree with the reviewer’s assessment. Accordingly, throughout the introduction, we have changed the structure of the introduction, and also added more references to explain our research objective. We found there are rare studies to explore the immediate and short-term effect on executive function, and this knowledge will allow us to build a framework for future research into high-efficiency approaches for increasing EF in a variety of people.

Comment 2. material and method

Comment 2.1 the calculation of sample size is not reported.

Answer: Thank you for this suggestion. We used the G*power to calculate the sample size, the sample size is 45. This work was done in a small sample size due to difficulty in recruiting subjects during the COVIN-19 period. our sample size only included 46 participants. we will added the calculation of sample size this part in the first paragraph in 2.1 Participants.

Comment 2.2 exclusion criteria are not included on: diet, sleeping, level of physical exercise practice, academic level, bilingualism, socioeconomic level, etc. that may interfere with the results obtained.

Answer: Thank you for this suggestion. We added more inclusion criteria in 2.1 participants to describe the participants in this study, and gave more detail in sleeping, the level of physical activity, the ability of education which can influent the cognitive performs.

Changes: page 2, 2.methods, 2.1 participants

Comment 2.2 how the exercise intensity of 60~70% is justified.

Answer: Thank you for pointing this out. previously, many researches has reported the acute aerobic exercise with moderate intensity improve executive function significantly.

[1].Kaminsky, L. A. (2014). ACSM's Guidelines for Exercise Testing and Prescription

[2]Tsukamoto, H., Takenaka, S., Suga, T., Tanaka, D., Takeuchi, T., Hamaoka, T., . . . Hashimoto, T. (2017). Impact of Exercise Intensity and Duration on Postexercise Executive Function. Med Sci Sports Exerc, 49(4), 774-784. <https://doi.org/10.1249/mss.0000000000001155>

[3]Effects of Acute Exercise Duration on the Inhibition Aspect of Executive Function in Late Middle-Aged Adults.We added explanation about the intensity and duration of the aerobic exercise protocol and some references in 2 methods 2.2.1 Aerobic exercise intervention.

Comment 2.3 it is necessary to specify the model of the cycle ergometer, the power that it develops,….

Answer: Thank you for pointing this out. We added the model of the bicycle ergometer, and we also added more information about the heart rate monitor.

Changes: all this information has added in 2.2.1 aerobic exercise intervention.

Comment 2.4 why does they always perform the same sequence of cognitive task?

Answer: Thank you for pointing this out. According to previous studies, four cognitive training tasks were designed in this study. Since the block of each task only appeared once, the subjects would complete these fore cognitive training tasks according to the difficulty of the task. (refrence: Rezola-Pardo, C., Arrieta, H., Gil, S. M., Yanguas, J. J., Iturburu, M., Irazusta, J., Rodriguez-Larrad, A. (2019). A randomized controlled trial protocol to test the efficacy of a dual-task multicomponent exercise program in the attenuation of frailty in long-term nursing home residents: Aging-ON(DUAL-TASK) study. BMC Geriatr, 19(1), 6. <https://doi.org/10.1186/s12877-018-1020-z>)

Comment 2.5 have you considered that the same sequence in the cognitive task can have an influence on the results?

Answer: Thank you for pointing this out. I am not very sure you are talking about the sequence of these training cognitive test or these three cognitive tasks for the executive function measure. For the training task, they only appeared once, and did not appear again, so we don’t think it can have influence on the results. But if you are talking about the three tasks for executive function test, we think it may had some influence on the results.

Comment 2.6 it is very likely that there is an effect on the outcome of cognitive tasks due to the progressive error. How was this effect controlled for?

Answer: Thank you for point this out. In this study, we tried to controlled all the progressive errors. At first, all the participants had been told the rules of each task, and they also had the practice trials to make sure they totally understand how to complete these tasks.

Comment 2.7 the effect size calculation should be included as well as data on the statistical power achieved and for each variable.

Answer: Thank you for this suggestion. We added more detail to describe the data in result. We added the mean difference and 95%IC.

Comment 3 the objective of the study is not well defined.

Answer: we appreciate the reviewer’s feedback. the objective of the study was further elaborated in the background.

Comment 4 minor corrections.

Comment 4.1 improve the writing of the document.

Answer: Thanks for your comment. We tried our best to improve the manuscript and made some change to the manuscript. This change will not influence the content and framework of the paper. And here we did not list the changes. This manuscript was edited by a native English speaker with a PhD in neuroscience.

Comment 4.2 correct misspelling.

Answer: Thank you for pointing this out. The “stoop” has been corrected on the table 1.

Comment 4.3 correct typos.

Answer: We are sorry for spelling mistakes and grammatical errors caused by our carelessness. In the revised version, we have made significant efforts to remove the mistakes and errors and improved writing. All the errors you picked and recommendations you proposed are greatly helpful for us to polish our manuscript. We appreciate your elaborate efforts in reviewing. Thank you very much!

We do appreciate your work in reviewing this paper. We hope these careful changes can make this paper more scientific and readable, and we really hope you can reconsider acceptance of this paper, because it really stands for our efforts and do contribute to the research of sports science.

Sincerely yours,

Hua Liu.

Capital University of Physical Education and Sports,

Email: liuhua@cupes.edu.cn
